# The Genomic Architecture of Fowl Typhoid Resistance in Commercial Layers

**DOI:** 10.3389/fgene.2018.00519

**Published:** 2018-11-19

**Authors:** Androniki Psifidi, Kay M. Russell, Oswald Matika, Enrique Sánchez-Molano, Paul Wigley, Janet E. Fulton, Mark P. Stevens, Mark S. Fife

**Affiliations:** ^1^The Roslin Institute and Royal (Dick) School of Veterinary Studies, The University of Edinburgh, Midlothian, United Kingdom; ^2^Royal Veterinary College, University of London, Hatfield, United Kingdom; ^3^Department of Infection Biology, Institute for Infection and Global Health, University of Liverpool, Neston, United Kingdom; ^4^Hy-Line International, Dallas Center, IA, United States; ^5^The Pirbright Institute, Surrey, United Kingdom

**Keywords:** fowl typhoid, chicken, layers, disease outbreak, GWAS, pathway

## Abstract

*Salmonella enterica* serovar Gallinarum causes devastating outbreaks of fowl typhoid across the globe, especially in developing countries. With the use of antimicrobial agents being reduced due to legislation and the absence of licensed vaccines in some parts of the world, an attractive complementary control strategy is to breed chickens for increased resistance to *Salmonella*. The potential for genetic control of salmonellosis has been demonstrated by experimental challenge of inbred populations. Quantitative trait loci (QTL) associated with resistance have been identified in many genomic regions. A major QTL associated with systemic salmonellosis has been identified in a region termed *SAL1*. In the present study, two outbreaks of fowl typhoid in 2007 and 2012 in the United Kingdom were used to investigate the genetic architecture of *Salmonella* resistance in commercial laying hens. In the first outbreak 100 resistant and 150 susceptible layers were genotyped using 11 single nucleotide polymorphism (SNP) and 3 microsatellite markers located in the previously identified *SAL1* region on chromosome 5. From the second outbreak 100 resistant and 200 susceptible layers, belonging to a different line, were genotyped with a high-density (600 K) genome-wide SNP array. Substantial heritability estimates were obtained in both populations (*h*^2^ = 0.22 and 0.26, for the layers in the first and second outbreak, respectively). Significant associations with three markers on chromosome 5 located close to *AKT1* and *SIVA1* genes, coding for RAC-alpha serine/threonine protein kinase, and the CD27-binding protein *SIVA1*, respectively, were identified in the first outbreak. From analysis of the second outbreak, eight genome-wide significant associations with *Salmonella* resistance were identified on chromosomes 1, 6, 7, 11, 23, 24, 26, 28 and several others with suggestive genome-wide significance were found. Pathway and network analysis revealed the presence of many innate immune pathways related to *Salmonella* resistance. Although, significant associations with SNPs located in the *SAL1* locus were not identified by the genome-wide scan for layers from the second outbreak, pathway analysis revealed P13K/AKT signaling as the most significant pathway. In summary, resistance to fowl typhoid is a heritable polygenic trait that could possibly be enhanced through selective breeding.

## Introduction

*Salmonella enterica* serovar Gallinarum causes a systemic bacterial disease mainly in adult poultry known as fowl typhoid. Outbreaks of this disease can have huge financial consequences with infected flocks having reduced egg production and a high percentage of mortality (Shivaprasad, 2000; Barrow and Freitas Neto, 2011). Regulations across the European Union compel poultry producers to control *Salmonella* in their layer and broiler breeder flocks. For example, in the United Kingdom, the Poultry Health Scheme routinely tests farms for the presence of *S.* Gallinarum resulting in rare occurrence of the disease after a prolonged control strategy (Poultry Health Scheme Handbook, 2013; Wigley, 2017). Despite such control measures, some outbreaks have been reported in recent years for both caged layers and backyard flocks in the United Kingdom indicating that outbreaks do still occur with devastating effects (Cobb et al., 2005; Parmar and Davies, 2007). More worrying, fowl typhoid has re-emerged in recent years in developing countries that have also established sanitary measures and official programs to prevent and control the disease. However, the disease remains endemic with cyclic or seasonal outbreaks related mainly to disease management (Revolledo, 2018). Therefore, a pressing need exists for complementary strategies to control the disease (Barbour et al., 2015; Guo et al., 2016; Celis-Estupinan et al., 2017; Pal et al., 2017; Weerasooriya et al., 2017).

Genetic selection for birds resistant to *S.* Gallinarum has been seen as an attractive solution for the control of fowl typhoid since the 1930’s (Lambert and Knox, 1932). Inbred chicken lines have been described that exhibit heritable differences in resistance to systemic salmonellosis, including following oral *S*. Gallinarum inoculation or intravenous administration of *S.* Typhimurium (Bumstead and Barrow, 1993; Mariani et al., 2001). These lines have been extensively studied over the past 35 years, and crosses between these lines have been used to identify quantitative trait loci (QTL) for *Salmonella r*esistance. A region on chromosome 5, termed *SAL1*, has been identified in multiple independent studies as having a protective role against systemic salmonellosis in the chicken (Mariani et al., 2001; Kaiser and Lamont, 2002; Tilquin et al., 2005; Calenge et al., 2010; Redmond et al., 2011). We refined the *SAL1* major QTL by mapping resistance in a 6th generation backcross with inbred lines 6_1_ (resistant) and 15I (susceptible) using a high-density SNP panel (Fife et al., 2009). The refined *SAL1* region contains 14 genes with some noticeable candidates that have previously been linked with *Salmonella* resistance in other species, such as the RAC-alpha serine/ threonine protein kinase homolog, *AKT* (Fife et al., 2009). It is noteworthy that distinct QTL have been associated with enteric carriage of *S*. Typhimurium (Fife et al., 2011).

The present study builds on and extends our previous studies in inbred lines, aiming to dissect the genomic architecture of fowl typhoid resistance using two different United Kingdom commercial layer populations which suffered from natural outbreaks of fowl typhoid. We conducted variance component analyses to estimate genetic parameters and genomic association studies to identify genomic regions controlling fowl typhoid resistance. We also performed gene enrichment and pathway analyses to identify candidate genes within the relevant genomic regions.

## Materials and Methods

### Ethics Statement

All animal experiments were conducted in accordance with the revised Animals (Scientific Procedures) Act 1986 (project license PPL40/3652) with the approval of the local Ethical Review Body.

### Study Population

Two different commercial laying hen populations suffering from two separate *S.* Gallinarum outbreaks of fowl typhoid, in 2007 and 2012 in the United Kingdom, were used in this study. From the first outbreak, blood and liver samples from 250 layers (150 susceptible and 100 resistant) were collected.

The second outbreak affected a layer farm with 375,000 birds. While most of the infected birds succumbed to infection, about 0.1% of the birds showed some level of resistance, with only mild clinical signs. Ultimately all remaining birds were culled on humane grounds, to prevent further spread of infection. From this outbreak, blood, spleen, and liver samples were collected from 300 layers (200 susceptible and 100 resistant). Three liver samples were collected from each bird, one in tissue storage reagent RNA*later*^®^, one in formalin for histological analysis, and one in phosphate-buffered saline (PBS) for enumeration of viable bacteria.

The collection of samples was performed by qualified veterinarians: samples were collected from birds raised in the same pens; live birds were culled and classified based on the observed pathology (lesions in liver, spleen, or ovary) as resistant or susceptible. Susceptible birds had extensive pathology implying potential death from lesions in the next 24 h. Resistant birds had no overt gross lesions on post mortem examination, with limited clinical signs.

For the first outbreak prevalence data was unavailable. For the second outbreak, the rate of infection varied between the six poultry houses on the affected premises. Levels of mortality consistent with clinical signs of fowl typhoid were recorded for the second outbreak with peak levels at approximately 3000 birds per day across the farm. Toward the end of the outbreak approximately 33% of birds had succumbed to infection. Birds for this study were sampled from the poultry house with the highest reported prevalence.

### Phenotyping

For the first outbreak the trait was binary [0/1, case (susceptible)-control (resistant)]. For the second outbreak *S.* Gallinarum load in liver was determined in colony-forming units (CFU)/gram as described previously (Mariani et al., 2001). Briefly, liver samples in PBS were weighed and homogenized in an equal v/w of PBS. The homogenized liver tissue was serially diluted and plated on Modified Brilliant Green Agar (Oxoid, United Kingdom), incubated overnight, and the numbers of bacterial colonies were counted. The number of CFU/g was log transformed in order to normalize the distribution. The trait for the second outbreak was analyzed both using continuous as well as binary phenotypes.

### Histology and Assessment of Pathogenicity

Histological analyses were performed on liver and spleen samples from birds from the second outbreak. Samples of liver and spleen were fixed in formalin, paraffin-wax embedded then cut and stained with haemotoxylin and eosin by the Department of Veterinary Pathology, University of Liverpool. Tissues were observed and analyzed blind as described previously (Parsons et al., 2013).

Assessment of pathogenicity of the strain isolated from the second outbreak in an experimental infection model was made in comparison with two well characterized *S.* Gallinarum isolates SG9 and 287/91 (Jones et al., 2001), as described previously (Langridge et al., 2015). Briefly, groups of five 3-week-old *Salmonella*-free commercial brown egg layer chickens (Lohmann Brown) were infected orally with 10^8^ CFU of each of the *S.* Gallinarum isolates or remained as an uninfected control. At 6 days post challenge all birds were killed and at post mortem examination the spleen, liver, and caecal contents were removed for enumeration of viable *Salmonella* on selective Modified Brilliant Green Agar (Oxoid, United Kingdom) as detailed previously (Langridge et al., 2015).

### Genotyping

All the birds from the first outbreak were genotyped using 11 custom-made SNP and 3 microsatellites markers located in the previously identified *SAL1* region on chromosome 5 (Fife et al., 2009). A full list of these markers is displayed in Supplementary Table S1. All the birds from the second outbreak were genotyped with the 600 K high density genome-wide SNP array (Affymetrix^®^ Axiom^®^ HD) (Kranis et al., 2013).

### Heritability Analyses

Genetic parameters were estimated for *S.* Gallinarum resistance for the first and the second outbreak using a mixed linear univariate model that included the population principal components (for the second outbreak only) as a covariate effect, and the random effect of the individual bird. Genetic relationships between birds were calculated based on SNP genotypes using the genome-wide efficient mixed model association (GEMMA) algorithm (Zhou and Stephens, 2014) and included in the analyses. For the second outbreak the continuous phenotypes were used to estimate the variance components. The heritability of each trait was calculated as the ratio of the additive genetic to the total phenotypic variance. All above analyses were performed separately for each outbreak using the ASReml 4.0 software (Gilmour et al., 2009).

### Genomic Association Analyses

#### Single-Marker Genomic Association Analyses

For the first outbreak a single marker association analysis where the SNP genotype was fitted as a fixed effect and the genomic relatedness matrix was fitted as a random polygenic effect was performed using ASReml 4.0 software (Gilmour et al., 2009).

Data from the second outbreak were analyzed using two genome-wide association methodologies. Briefly, either a single SNP or a group of SNPs in sets of windows/ regions-using a regional heritability mapping approach (RHM)- were fitted as fixed effects.

The SNP genotype data were subjected to quality control measures using PLINK v1.09 (Purcell et al., 2007): minor allele frequency >0.05, call rate >95% and Hardy–Weinberg equilibrium (*P* > 10^−6^). After quality control, 297,560 SNP markers remained for further analysis. Positions of SNP markers were obtained using the Gal-gal5 assembly in Ensembl Genome Browser ^1^.

Population stratification was investigated using a genomic relatedness matrix generated from all individuals. This genomic relatedness matrix was converted to a distance matrix that was used to carry out classical multidimensional scaling analysis (MSA) using the GenABEL package of R (Aulchenko et al., 2007), to obtain its principal components.

The GEMMA algorithm (Zhou and Stephens, 2014) was used to perform GWAS analyses using a standard univariate linear mixed model in which the first four principal components were fitted as covariate effects to adjust for population structure and the genomic relatedness matrix among individuals was fitted as a polygenic effect. After Bonferroni correction for multiple testing, significance thresholds were *P* ≤ 1.68 × 10^−7^ and *P* ≤ 3.36 × 10^−6^ for genome-wide significant (*P* ≤ 0.05) and suggestive (namely one false positive per genome scan) levels, respectively, corresponding to −log_10_(P) of 6.77 and 5.47. The Chi-square (χ^2^) test was implemented to validate the GWAS results. A *P*-value for each comparison (expected vs. observed values) was estimated based on the χ^2^ statistics value for two degrees of freedom. The significance threshold was set at *P* ≤ 0.05. The extent of linkage disequilibrium (LD) between significant SNPs located on the same chromosome regions was calculated using the *r*-square statistic of PLINK v1.09 (Purcell et al., 2007).

#### Regional Heritability Mapping

The RHM approach was used to analyse data from the second outbreak fitting genomic regions of 20 SNPs in sliding “windows” along each chromosome. RHM analyses were performed using the DISSECT software (Canela-Xandri et al., 2015) fitting the same fixed effects as the ones used in the single SNP GWAS described above. The significance of genomic regions was assessed with the likelihood ratio test statistic, which was used to compare the RHM model where both the whole genome and a genomic region were fitted as random effects against the base model that excluded the latter effect. A total of 14,878 regions were tested across the genome. After the adjustment, using Bonferroni correction, for multiple testing significance thresholds were *P* ≤ 3.37 × 10^−6^ and *P* ≤ 6.72 × 10^−5^ for genome-wide (*P* ≤ 0.05) and suggestive (namely one false positive per genome scan) levels, respectively, corresponding to −log_10_(P) of 5.47 and 4.17.

### SNP and Candidate Region Annotation

All significant SNPs identified in the GWAS for the second *S.* Gallinarum outbreak were mapped to the reference genome and annotated by using the variant effect predictor^2^ tool within the Ensembl database and the Gal-gal5 assembly. Moreover, the genes that were located 100 kb upstream and downstream of the significant SNPs were also annotated using the BioMart data mining tool^3^ and the Gal-gal5 assembly. We chose these 200 kb windows based on the average LD in commercial populations (less than 1 cM on average; Andreescu et al., 2007) and the fact that the chicken genome contains 250 kb per cM on average (International Chicken Genome Sequencing Consortium, 2004). This allowed us to catalog all the genes that were located in the vicinity of the identified significant SNPs and to create gene lists that contained the genes in the vicinity of all the significant SNPs identified for fowl typhoid resistance.

### Pathway, Network and Functional Enrichment Analyses

Identification of potential canonical pathways and networks underlying the candidate genomic regions associated with resistance to the second *S.* Gallinarum outbreak was performed using the Ingenuity Pathway Analysis (IPA) program ^4^. IPA constructs multiple possible upstream regulators, pathways, and networks that serve as hypotheses for the biological mechanism underlying the phenotypes based on a large-scale causal network derived from the Ingenuity Knowledge Base. Then, IPA infers the most suitable pathways and networks based on their statistical significance, after correcting for a baseline threshold (Krämer et al., 2014). The IPA score in the constructed networks can be used to rank these networks based on the *P*-values obtained using Fisher’s exact test [IPA score or *P*-score = −log_10_(*P*-value)].

The gene list for *S.* Gallinarum resistance was also analyzed using the Database for Annotation, Visualization and Integrated Discovery (DAVID; Dennis et al., 2003). In order to understand the biological meaning behind these genes, gene ontology (GO) was determined and functional annotation clustering analysis was performed. The *Gallus gallus* background information is available in DAVID and was used for the analysis. The enrichment score (ES) of the DAVID package is a modified Fisher exact *P*-value calculated by the software, with higher ES reflecting more enriched clusters. An ES greater than 1 means that the functional category is overrepresented.

## Results

### Descriptive Statistics of Phenotypes

A mean three-log difference of liver *S.* Gallinarum viable counts between the resistant (average: 4.4 log_10_CFU/gr, standard deviation: 1.66) and the susceptible (average: 7.4 log_10_CFU/gr, standard deviation: 0.77) birds from the second outbreak was detected, consistent with the pathology results. The maximum of liver count measured was 8.45 log_10_ CFU/gr, while in 34 samples no viable *S.* Gallinarum was detected (minimum).

### Histology and Assessment of Pathogenicity

**FIGURE 1:**
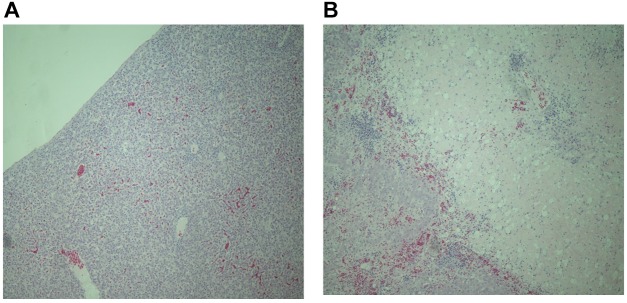
Representative haematoxylin and eosin stained sections of liver tissue from resistant **(A)** and susceptible **(B)** chickens from the second outbreak (magnification × 400). The liver of susceptible birds show extensive necrotic tissue damage and massive and widespread influx of inflammatory cells whereas resistant birds show smaller defined loci of inflammation.

As many samples were autolysed or degraded detailed scoring was not possible. However, analysis of tissues from six resistant and nine susceptible birds where the sample was not compromised, showed patterns of pathology similar with the ones previously described following experimental infection of resistant and susceptible inbred lines with *S.* Gallinarum (Wigley et al., 2002). Resistant birds showed signs of inflammation, largely restricted to specific foci in the liver (Figure 1A) and general inflammation in the spleen. In contrast susceptible birds showed greater levels of inflammation and large areas of necrotic damage in the liver (Figure 1B), with a high degree of inflammatory cell influx into the spleen with thickening of the splenic capsule and some areas of necrosis. These findings are consistent with observations in inbred lines exhibiting differential resistance following experimental infection (Mariani et al., 2001).

In experimental infection studies, a clonal isolate from the second outbreak was recovered in equivalent or greater numbers from the spleen and liver of orally challenged birds than 287/91 or SG9 (Supplementary Figure S1). This fulfills Koch’s postulates and the outbreak strain may be considered typical of other *S.* Gallinarum strains in the pathology it elicits. None of the isolates were detected in the caecal contents at the time of post mortem examination.

### Single-Marker Genomic Association Studies

Similar moderate heritability estimates for *S.* Gallinarum resistance were derived for both layer populations in the first (*h*^2^ = 0.22 ± 0.01) and second (*h*^2^ = 0.26 ± 0.14) outbreaks.

Seven markers located in the *SAL1* locus on chromosome 5 were found to have a significant (*P* < 0.05) association with *S.* Gallinarum resistance in the layer population affected by the first outbreak. Details of the significant markers identified are presented in Table 1.

**Table 1:** List of SNPs associated with fowl typhoid resistance in the layer population from the first outbreak.

SNP Name	Chromosome	Position	*P*-value
SNP7	5	50401216	0.005
SNP94	5	50471836	0.002
SNP215	5	51415477	0.026
**SNP197**	5	51685833	0.006
**SNP200**	5	51686240	<0.001
**SNP201**	5	51686379	<0.001
**SNP202**	5	51739862	0.047

*SNP markers in bold are spanning the AKT1 gene.*

Multidimensional scaling analysis revealed four substructure principal components in the layer population affected by the second outbreak, which were subsequently included in the GWAS model to correct results for population stratification.

**FIGURE 2:**
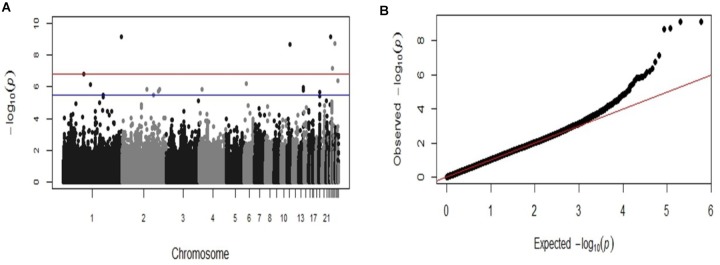
Manhattan plot and Q-Q plot displaying the GWAS results from the second fowl typhoid outbreak (continuous phenotypes). **(A)** Genomic location is plotted against –log_10_(P) in the Manhattan plot. Genome-wide (*P* < 0.05) and suggestive genome-wide thresholds are shown as red and blue lines, respectively. **(B)** Q–Q plot of observed *P*-values against the expected *P*-values for *Salmonella* Gallinarum liver load (log-transformed CFU of *S.* Gallinarum per gram of liver).

GWAS analysis identified six SNP markers genome-wide significantly associated with the log-transformed liver load of *S.* Gallinarum in layers from the second outbreak on chromosomes 1, 11, 23, 24, and 26 (*P*-values 7.36 × 10^−10^ to 1.63 × 10^−7^) (Table 2). Additionally, 14 SNPs crossing the suggestive genome-wide significant threshold were identified on chromosomes 1, 2, 4, 6, 13, 19, 24, and 28 (Table 2). The Manhattan plot and the Q-Q plot for the GWAS results are displayed in Figures 2A,B.

**Table 2:** List of SNPs associated with fowl typhoid resistance in the layers from the second outbreak.

Phenotype	SNP name	Chr	Position	*P*-value
**Continues**				
	**Affx-50313880**	**1**	**194531778**	**7.368E-10**
	**Affx-51098463**	**23**	**5081233**	**7.468E-10**
	**Affx-51148005**	**26**	**5073473**	**1.852E-09**
	**Affx-50516977**	**11**	**11072620**	**2.042E-09**
	**Affx-51116866**	**24**	**4720171**	**7.372E-08**
	**Affx-50405629**	**1**	**67549126**	**1.636E-07**
	Affx-51177949	28	3758677	4.249E-07
	Affx-51686897	6	8299315	6.298E-07
	Affx-50447114	1	91550805	6.893E-07
	Affx-50617622	13	16337564	1.082E-06
	Affx-51370634	4	10316583	1.373E-06
	Affx-50841906	2	127840521	1.379E-06
	Affx-50988352	2	85262253	1.468E-06
	Affx-50617378	13	16238356	1.572E-06
	Affx-50617564	13	16313839	1.572E-06
	Affx-50832761	2	122511846	1.865E-06
	Affx-50780736	19	4130929	2.219E-06
	Affx-50193882	1	133009585	3.339E-06
	Affx-50808404	2	107286660	3.424E-06
	Affx-51107231	24	2183253	3.424E-06
**Binary**				
	**Affx-51177949**	**28**	**3758677**	**2.414E-12**
	**Affx-51643081**	**6**	**20430378**	**8.818E-09**
	**Affx-51739265**	**7**	**31474122**	**1.083E-08**
	**Affx-50405629**	**1**	**67549126**	**2.015E-07**
	Affx-50538456	11	19872676	2.724E-07
	Affx-51148005	26	5073473	2.808E-07
	Affx-51088276	23	2561442	6.146E-07
	Affx-50414020	1	71999883	9.84E-07
	Affx-51197199	3	107050735	1.026E-06
	Affx-50476276	10	14891444	1.75E-06
	Affx-51098463	23	5081233	2.67E-06

*P-value from genomic association study (genome-wide significant in bold, suggestive significance otherwise).*

**FIGURE 3:**
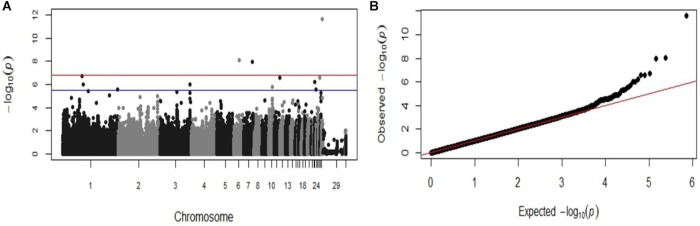
Manhattan plot and Q-Q plot displaying the GWAS results from the second fowl typhoid outbreak (binary (0/1) phenotypes). **(A)** Genomic location is plotted against −log_10_(P) in the Manhattan plot. Genome-wide (*P* < 0.05) and suggestive genome-wide thresholds are shown as red and blue lines, respectively. **(B)** Q–Q plot of observed *P*-values against the expected *P*-values for *Salmonella* Gallinarum resistance.

The same significant associations on chromosomes 1, 23, 26, and 28 were identified by the GWAS analysis when the data was re-analyzed as a binary (case-control) trait (Table 2), although the ranking of the SNPs based on the *P*-values were different. With the case-control analysis the association on chromosome 28 attained genome-wide significance (*P*-values 2.41 × 10^−12^). This approach identified also two new genome-wide significant associations on chromosomes 6 and 7 (*P*-values 8.81 × 10^−9^ to 1.08 × 10^−8^) and new suggestive associations with markers on chromosomes 1, 3, 10, 11, and 23 (Table 2). All the significant associations identified by the GWAS were also found to be significant (*P* < 0.05) in the chi-square analysis. The Manhattan plot and the Q-Q plot for the GWAS results from the case-control analysis are displayed in Figures 3A,B. Significant SNPs that were located on the same chromosome were not in LD with the exception of the markers located on chromosome 13 (*r*^2^ > 0.90).

### Regional Heritability Mapping

The RHM mapping confirmed the significant associations on chromosomes 1, 11, 23, 24, and 26 previously identified by the GWAS (Supplementary Table S2). Moreover, RHM detected two more suggestive significant associations on chromosomes 2 and 11. Details of the significant SNP windows are presented in Supplementary Table S2. The Manhattan plot and the Q-Q plot for the RHM results analysis are displayed in Supplementary Table S2.

### SNP and Candidate Region Annotation

All of the significant markers identified for the first outbreak were located in intronic, intergenic, upstream and downstream gene regions with the exception of one SNP (SNP7) which corresponds to a missense variant within the Creatine Kinase B (*CKB*) gene. Four of the significant SNP markers spanned the *AKT1* gene. All these SNPs are intronic variants for the *AKT1* and also upstream and downstream variants for one microRNA (gga-mir-1771). The candidate region for fowl typhoid on chromosome 5 contained 16 protein coding genes and 2 microRNAs (Supplementary Table S3).

Most of the significant SNPs identified by the GWAS analyses for the second outbreak were located in intronic (34%), intergenic (24%) and upstream and downstream gene (14%) regions. However, four of the SNPs were localized in exonic regions. Specifically, Affx-51116866 corresponds to a missense variant within the Cell Adhesion Molecule 1 (*CADM1*) gene; Affx-51148005 corresponds to a missense variant within the TATA-Box Binding Protein Associated Factor 8 (*TAF8*) gene; Affx-51686897 corresponds to a missense variant within the AT-Rich Interaction Domain 5B (*ARID5B*) gene; Affx-51177949 corresponds to a synonymous variant within the Growth Differentiation Factor 3 (*GDF3*) gene. The above mentioned missense variants had a predicted moderate impact.

Most of the candidate regions for fowl typhoid resistance identified from the second outbreak contained multiple genes. In total 116 protein-coding genes and 4 microRNAs identified across the QTL regions for the second outbreak (Supplementary Table S3).

### Pathway, Network and Functional Enrichment Analyses

**FIGURE 4:**
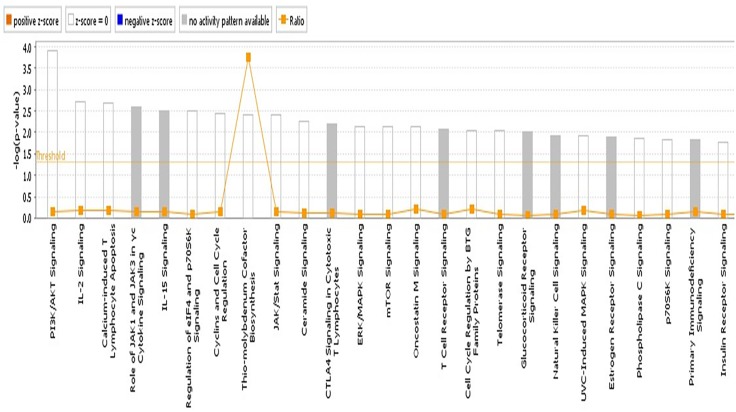
Pathway analysis using the IPA software. The most highly represented canonical pathways derived from genes located within the candidate regions for fowl typhoid resistance in the layer population affected by the second outbreak. The solid yellow line represents the significance threshold. The line joining squares represents the ratio of the genes represented within each pathway to the total number of genes in the pathway.

**FIGURE 5:**
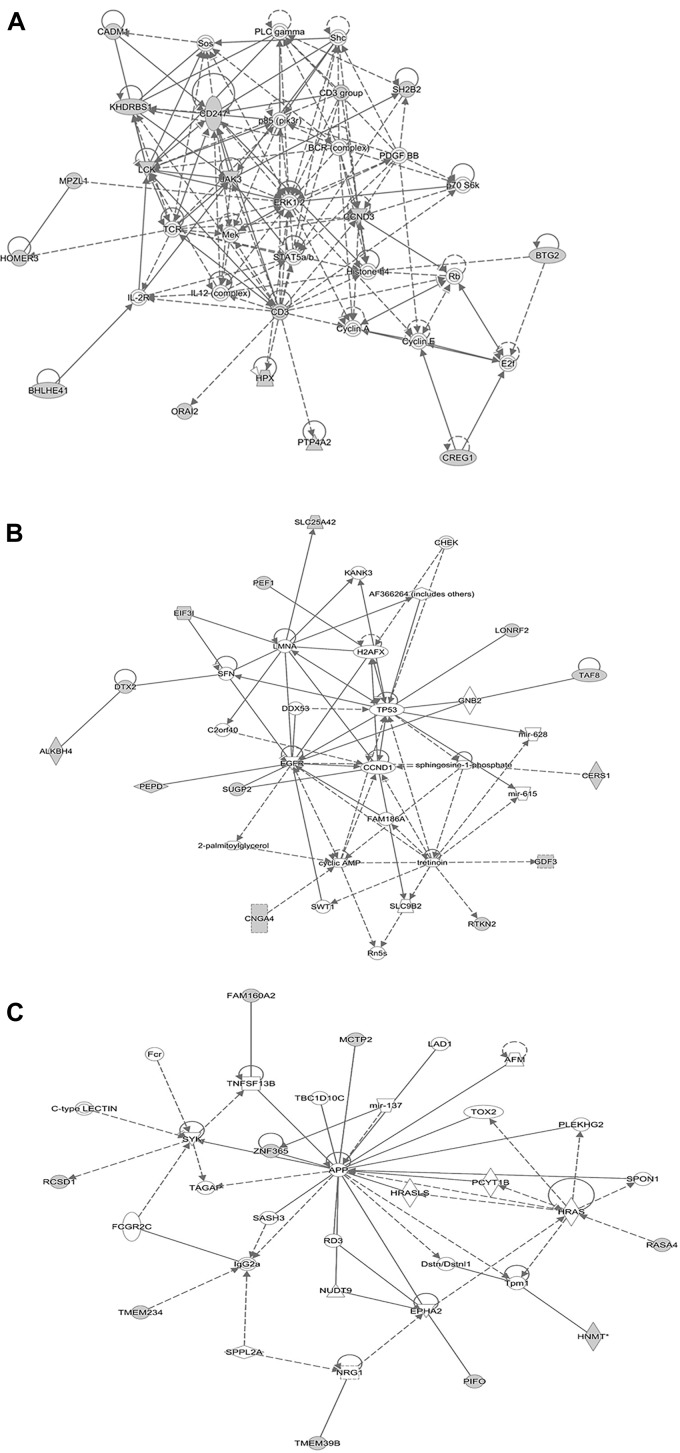
Network analysis using the IPA software. The three networks (**A** related to cellular development, hematological system development and function, hematopoiesis), (**B** related to cell to cell signaling and interaction, cellular compromise, cellular development), and (**C** related to cell cycle, cell death and survival, cellular development) illustrate molecular interactions between products of candidate genes selected from the QTL regions for fowl typhoid resistance in the layer population affected by the second outbreak. Arrows with solid lines represent direct interactions and arrows with broken lines represent indirect interactions. Genes with white labels are those added to the IPA analysis because of their interaction with the target gene products.

We reasoned that the corresponding QTL regions may contain genes contributing to a common pathway associated with *S.* Gallinarum resistance. We therefore identified the sets of annotated genes lying within the QTL intervals identified for the second outbreak and sought evidence of gene set enrichment. These genes were enriched for pathways involved in immune responses, both innate and adaptive, and cell-cycle regulation (Figure 4). The most enriched pathway was related to the P13K/AKT signaling. Moreover, three networks of molecular interactions related to cell death and survival, and cell cycle, humoral immune response, hematological system development and function, and hematopoiesis were constructed using the list of genes in the candidate regions (Figure 5).

Functional annotation clustering analysis revealed the presence of enriched gene clusters related to protein kinase binding (E.S = 2.35, genes in the cluster: *PRKRIP1, CCND3, YWHAG, HDAC1*), positive regulation of immune system processes (E.S = 1.7, genes in the cluster: *CD247, SH2B2, CADM1, HPX, LCK*), hematopoiesis and immune system development (E.S = 1.1, genes in the cluster: *CEBPA, CEBPG, LCK, RTKN2*).

## Discussion

Our study set out to investigate the genetic basis of fowl typhoid resistance in commercial layers. Using samples from two natural disease outbreaks, we detected heritable genetic variation and identified genomic regions associated with resistance to the disease in two different layer populations. Putative candidate genes, canonical pathways and networks involved in the underlying molecular mechanisms of fowl typhoid resistance were also identified.

In terms of phenotype, there was on average a 3 Log_10_ difference in the recovery of viable *S*. Gallinarum between resistant and susceptible birds from the second outbreak and differences in pathology that are consistent with those observed following experimental infection of inbred lines that exhibit heritable differences in resistance following oral *S.* Gallinarum or intravenous *S.* Typhimurium inoculation (Bumstead and Barrow, 1993; Mariani et al., 2001). Although much of the QTL-based mapping of the *SAL1* locus in inbred lines used intravenous infection of day old chicks with *S*. Typhimurium, the phenotype of resistance to experimental fowl typhoid is strongly expressed in older birds (Bumstead and Barrow, 1993) with quantitative differences of 3–4 Log_10_ CFU per gram of liver tissue between resistant and susceptible lines found 8 days after oral challenge with *S*. Gallinarum in 3-week-old birds (Wigley et al., 2002). Therefore, the pathology, phenotyping and histological results of the present study conducted in commercial layers are consistent with previous findings in inbred lines for fowl typhoid infection.

In addition, the present study provided further evidence for the role of *SAL1* locus in *Salmonella* resistance. *AKT1* is a promising candidate gene of this QTL region as the protein is known to be activated by *Salmonella* and to promote intracellular net replication of the bacteria in mammalian cells (Steele-Mortimer et al., 2000; Kuijl et al., 2007). In the first outbreak the markers with the most significant association with the fowl typhoid spanned the *AKT1* gene. Although, significant associations with SNPs located in the *SAL1* locus were not identified by the genome-wide scan for layers from the second outbreak, pathway analysis revealed that the P13K/AKT signaling as the most significant pathway, implying that *AKT* pathway might play a role in *Salmonella* resistance. It is possible that other genes that are part of the P13/AKT pathway such as *JAK3, KRAS, GYS2, PPP2CA, YWHAG* might contribute to fowl typhoid resistance in the layers of the second outbreak since they belong to a different selection line and SNP markers proximal to these genes were identified in the GWAS analysis. Therefore, although the underlying mechanism might be similar, the causative mutation(s) might be different in the two populations. In addition, the phase of LD between the SNP markers and the causative mutation(s) might be different in the two different layer populations. *AKT* is a serine/threonine kinase that modulates multiple processes, in particular apoptosis, cell proliferation, and development (Hers et al., 2011). Depending on the cell type and stage of infection, apoptosis may play both positive and negative roles in control of *Salmonella* infection (Fink and Cookson, 2007). Nevertheless, the involvement of the other striking candidate gene, the CD27-binding protein *SIVA1*, in fowl typhoid resistance could not be excluded since the two candidate genes are in close proximity and significant markers were detected on either sides of these genes. *SIVA1* is a pro-apoptotic factor that induces cell death via a caspase-dependent pathway in human and murine cells (Prasad et al., 1997; Py et al., 2004). It has been also proposed that differences in the expression or function of *SIVA1* in the progeny of advanced inter-cross chicken lines may explain differences in the ability of heterophils from such birds to release heterophil extracellular traps via an apoptosis-like pathway (Redmond et al., 2011).

This is the first study, to our knowledge, that aimed to dissect the genetic architecture of fowl typhoid resistance using data of natural disease outbreaks. However, there are many previous genetic studies of systemic salmonellosis, *Salmonella* enteric carriage, carrier-state and antibody responses based on challenge experiments of *S.* Enteritidis and *S.* Typhimurium in crosses of inbred, and crosses of inbred with commercial chicken lines. Interestingly, many of the previously identified QTLs are overlapping or are in close proximity with the ones identified in the present study. The two QTLs we identified on chromosome 1 at position 67.5 and 91.5 Mb are closely located; the former with one identified in inbred lines for cloacal bacterial burden after oral challenge with *S.* Enteritidis (Tilquin et al., 2005) and the latter with one identified in broiler crosses for spleen bacterial burden after intra-oesophageal challenge (Kaiser and Lamont, 2002) and for vaccine response after subcutaneous challenge with *S.* Enteritidis (Kaiser et al., 2002). The QTLs on chromosome 1 (194.5 Mb) and chromosome 11 (20 Mb) overlap with QTLs found in inbred line crosses for carrier *Salmonella* state after oral challenge with *S.* Enteritidis (Calenge et al., 2011). Likewise, the QTL regions on chromosome 2 (122 Mb) and 4 overlap with previously identified QTLs for spleen bacterial burden after challenge with *S.* Enteritidis intra-oesophageal (Malek et al., 2004). The QTL on chromosome 3 overlaps with a QTL identified in advanced intercrosses of inbred lines with broilers for spleen bacterial burden after intra-oesophageal infection with *S.* Enteritidis (Hasenstein and Lamont, 2007). In the latter study, the gallinacin group of genes were considered good candidate genes for *Salmonella* resistance. The gallinacin-8 precursor (*AvBD8*) gene is also in close proximity with the significant marker identified on chromosome 3 in the present study. However, more studies are needed to confirm if this is the actual causative gene for this QTL. The QTLs that we identified on chromosomes 7, 19, 23, 24, and 26 are co-localized with previously identified QTLs in inbred chicken line crosses for *S.* Enteritidis caeca colonization after oral inoculation (Thanh-Son et al., 2012). Many immune genes (such as *LAT2/NTAL, TRAF3IP3, IRF6*) located within these QTL regions have been suggested as good candidate genes for *Salmonella* resistance.

The present study implemented a much higher density genome-wide genotyping platform compared to all the previous ones and was able to identify some novel QTLs. Moreover, two different approaches, GWAS and RHM, were implemented to further facilitate the QTL discovery. GWAS performs single marker analyses while RHM fits genomic regions of multiple SNPs as a single measure. Therefore, RHM has greater power compared to GWAS to identify loci where several alleles with small effects segregating. In addition, we implemented two different GWAS models, one using binary phenotypes and the other the continuous phenotypes. We used the binary phenotypes to be consistent with the phenotypes used to analyse the first outbreak, and the continuous ones to increase further the power of the study and overcome putative errors derived from misclassifications of cases and controls. The marker on chromosome 28 found to have the most significant association with fowl typhoid resistance, when the trait was analyzed as binary, is surrounded by many putative good candidate genes. Such genes related with immune response are the tyrosine-protein Janus kinase 3 (*JAK3*), the CREB regulator transcription coactivator 1 (*CRTC1*) and the cytokine receptor like factor 1 (*CRLF1*). The IPA analysis identified two canonical pathways related with JAK signaling among the most enriched pathways in this dataset: the JAK1 and JAK3 in the γc cytokine regulation signaling and the JAK-Stat signaling. In addition, the immune related network with the highest IPA score had as one of the central molecules the *JAK3* protein. The JAK signaling family of tyrosine kinases are involved in cytokine receptor-mediated intracellular signal transduction. Specifically, *JAK3* mediates essential signaling events in both innate and adaptive immunity and plays a crucial role in hematopoiesis during T-cells development (Yamaoka et al., 2004). Multiple markers on chromosome 13 were found to have a significant association with fowl typhoid resistance. These markers span the follistatin-related protein 4 precursor (*FSTL4*) gene which is related with calcium metabolism and transportation. However, in close proximity (<0.5 Mb), immune genes of interest such as the Interleukin 3 precursor (*IL-3*), Interleukin 5 precursor (*IL5*), and the Interferon Regulatory Factor 1 (*IRF1*), are located. The protein encoded by *IRF1* gene is a transcriptional regulator and tumor suppressor, serving as an activator of genes involved in both innate and acquired immune responses. The encoded protein activates the transcription of genes involved in the body’s response to viruses and bacteria, playing a role in cell proliferation, apoptosis, immune and DNA damage response (Yoshida and Azuma, 1992; Taniguchi et al., 1995). In addition, it is involved in the regulation of interferon (*IFN*) and IFN-inducible genes that have been reported to be involved in host resistance to *Salmonella* infection (Thanh-Son et al., 2012).

## Conclusion

We confirmed that resistance to fowl typhoid is a heritable complex polygenic trait. Co-localisation of many of the QTLs identified for fowl resistance with previous ones identified for systemic and enteric salmonellosis, and antibody responses implying that common underlying mechanisms of resistance to different *Salmonella* serovars segregating across chicken populations. These findings strengthen the interest of these regions for more refined analyses. According to our results breeding for enhanced fowl typhoid resistance in layers is possible. Although genomic selection is a valid approach to enhance disease resistance in chickens, as has been reported previously (Legarra et al., 2011), identification of the causative genes and mutations could expedite selection through different weighting of the validated selectable markers or precision breeding. However, further studies are needed to identify the causative genes and mutations.

## Data Availability Statement

All data are available at Figshare (doi: 10.6084/m9.figshare.7205702).

## Author Contributions

MF, MS conceived and designed the genetic studies of fowl typhoid and secured funding. AP and OM performed the genetic parameter analysis, collated and edited the genotyping data, and performed the single marker genomic analysis. AP and ES-M performed the regional heritability mapping. AP performed the pathway analysis and wrote the manuscript with input from KR. PW performed the histological analysis and experimental infections. MF, MS, JF, KR, and AP interpreted these results. All other co-authors provided manuscript editing and feedback. All authors read and approved the final manuscript.

## Conflict of Interest Statement

JF was employed by Hy-Line International. The remaining authors declare that the research was conducted in the absence of any commercial or financial relationships that could be construed as a potential conflict of interest.

## Footnotes

1 www.ensembl.org

2 http://www.ensembl.org/Tools/VEP

3 http://www.ensembl.org/biomart/martview/

4 www.ingenuity.com

## References

[B1] Andreescu C. Avendano S. Brown S. R. Hassen A. Lamont S. J. Dekkers J. C. (2007). Linkage disequilibrium in related breeding lines of chickens. *Genetics* 177 2161–2169. 10.1534/genetics.107.082206 17947400 PMC2219483

[B2] Aulchenko Y. S. Ripke S. Isaacs A. Van Duijn C. M. (2007). GenABEL: an R library for genome-wide association analysis. *Bioinformatics* 23 1294–1296. 10.1093/bioinformatics/btm108 17384015

[B3] Barbour E. K. Ayyash D. B. Alturkistni W. Alyahiby A. Yaghmoor S. Iyer A. (2015). Impact of sporadic reporting of poultry *Salmonella* serovars from selected developing countries. *J. Infect. Dev. Ctries.* 9 1–7. 10.3855/jidc.5065 25596565

[B4] Barrow P. A. Freitas Neto O. C. (2011). Pullorum disease and fowl typhoid–new thoughts on old diseases: a review. *Avian Pathol.* 40 1–13. 10.1080/03079457.2010.542575 21331943

[B5] Bumstead N. Barrow P. (1993). Resistance to *Salmonella* gallinarum, S. pullorum, and S. enteritidis in inbred lines of chickens. *Avian Dis.* 37 189–193. 10.2307/1591473 8452495

[B6] Calenge F. Kaiser P. Vignal A. Beaumont C. (2010). Genetic control of resistance to salmonellosis and to *Salmonella* carrier-state in fowl: a review. *Genet. Sel. Evol.* 42:11. 10.1186/1297-9686-42-11 20429884 PMC2873309

[B7] Calenge F. Vignal A. Demars J. Feve K. Menanteau P. Velge P. (2011). New QTL for resistance to *Salmonella* carrier-state identified on fowl microchromosomes. *Mol. Genet. Genomics* 285 237–243. 10.1007/s00438-011-0600-9 21279652

[B8] Canela-Xandri O. Law A. Gray A. Woolliams J. A. Tenesa A. (2015). A new tool called DISSECT for analysing large genomic data sets using a big data approach. *Nat. Commun.* 6:10162. 10.1038/ncomms10162 26657010 PMC4682108

[B9] Celis-Estupinan A. Batista D. F. A. Cardozo M. V. Secundo De Souza A. I. Rodrigues Alves L. B. Maria De Almeida A. (2017). Further investigations on the epidemiology of fowl typhoid in Brazil. *Avian Pathol.* 46 416–425. 10.1080/03079457.2017.1299922 28277779

[B10] Cobb S. P. Mcvicar C. M. Davies R. H. Ainsworth H. (2005). Fowl typhoid in caged layer birds. *Vet. Rec.* 157:268. 10.1136/vr.157.9.268 16127143

[B11] DennisG.Jr.ShermanB. T.HosackD. A.YangJ.GaoW.LaneH. C. (2003). DAVID: database for annotation, visualization, and integrated discovery. *Genome Biol.* 4:R60 10.1186/gb-2003-4-9-r6012734009

[B12] Fife M. S. Howell J. S. Salmon N. Hocking P. M. Van Diemen P. M. Jones M. A. (2011). Genome-wide SNP analysis identifies major QTL for *Salmonella* colonization in the chicken. *Anim. Genet.* 42 134–140. 10.1111/j.1365-2052.2010.02090.x 20579012

[B13] Fife M. S. Salmon N. Hocking P. M. Kaiser P. (2009). Fine mapping of the chicken salmonellosis resistance locus (SAL1). *Anim. Genet.* 40 871–877. 10.1111/j.1365-2052.2009.01930.x 20597881

[B14] Fink S. L. Cookson B. T. (2007). Pyroptosis and host cell death responses during *Salmonella* infection. *Cell. Microbiol.* 9 2562–2570. 10.1111/j.1462-5822.2007.01036.x 17714514

[B15] Gilmour A. R. Cullis B. R. Thompson R. (2009). *ASREML User Guide, Release 3.0*. Orange, NSW: NSW Department of Primary Industries.

[B16] Guo R. Geng S. Jiao H. Pan Z. Chen X. Jiao X. (2016). Evaluation of protective efficacy of a novel inactivated *Salmonella* Pullorum ghost vaccine against virulent challenge in chickens. *Vet. Immunol. Immunopathol.* 173 27–33. 10.1016/j.vetimm.2016.03.015 27090623

[B17] Hasenstein J. R. Lamont S. J. (2007). Chicken gallinacin gene cluster associated with *Salmonella* response in advanced intercross line. *Avian Dis.* 51 561–567. 10.1637/0005-2086(2007)51[561:CGGCAW]2.0.CO;2 17626484

[B18] Hers I. Vincent E. E. Tavare J. M. (2011). Akt signalling in health and disease. *Cell. Signal.* 23 1515–1527. 10.1016/j.cellsig.2011.05.004 21620960

[B19] International Chicken Genome Sequencing Consortium (2004). Sequence and comparative analysis of the chicken genome provide unique perspectives on vertebrate evolution. *Nature* 432 695–716. 15592404 10.1038/nature03154

[B20] Jones M. A. Wigley P. Page K. L. Hulme S. D. Barrow P. A. (2001). *Salmonella enterica* serovar Gallinarum requires the *Salmonella* pathogenicity island 2 type III secretion system but not the *Salmonella* pathogenicity island 1 type III secretion system for virulence in chickens. *Infect. Immun.* 69 5471–5476. 10.1128/IAI.69.9.5471-5476.2001 11500419 PMC98659

[B21] Kaiser M. G. Deeb N. Lamont S. J. (2002). Microsatellite markers linked to *Salmonella enterica* serovar enteritidis vaccine response in young F1 broiler-cross chicks. *Poult. Sci.* 81 193–201. 10.1093/ps/81.2.193 11873827

[B22] Kaiser M. G. Lamont S. J. (2002). Microsatellites linked to *Salmonella enterica* Serovar Enteritidis burden in spleen and cecal content of young F1 broiler-cross chicks. *Poult. Sci.* 81 657–663. 10.1093/ps/81.5.657 12033415

[B23] Krämer A. Green J. Pollard J. Tugendreich S. (2014). Causal analysis approaches in ingenuity pathway analysis. *Bioinformatics* 30 523–530. 10.1093/bioinformatics/btt703 24336805 PMC3928520

[B24] Kranis A. Gheyas A. A. Boschiero C. Turner F. Yu L. Smith S. (2013). Development of a high density 600K SNP genotyping array for chicken. *BMC Genomics* 14:59. 10.1186/1471-2164-14-59 23356797 PMC3598943

[B25] Kuijl C. Savage N. D. Marsman M. Tuin A. W. Janssen L. Egan D. A. (2007). Intracellular bacterial growth is controlled by a kinase network around PKB/AKT1. *Nature* 450 725–730. 10.1038/nature06345 18046412

[B26] Lambert W. V. Knox C. W. (1932). Selection for resistance to fowl typhoid in the chicken with reference to its inheritance. *Res. Bull.* 12:1.

[B27] Langridge G. C. Fookes M. Connor T. R. Feltwell T. Feasey N. Parsons B. N. (2015). Patterns of genome evolution that have accompanied host adaptation in *Salmonella*. *Proc. Natl. Acad. Sci. U.S.A.* 112 863–868. 10.1073/pnas.1416707112 25535353 PMC4311825

[B28] Legarra A. Calenge F. Mariani P. Velge P. Beaumont C. (2011). Use of a reduced set of single nucleotide polymorphisms for genetic evaluation of resistance to *Salmonella* carrier state in laying hens. *Poult. Sci.* 90 731–736. 10.3382/ps.2010-01260 21406356

[B29] Malek M. Hasenstein J. R. Lamont S. J. (2004). Analysis of chicken TLR4, CD28, MIF, MD-2, and LITAF genes in a *Salmonella* enteritidis resource population. *Poult. Sci.* 83 544–549. 10.1093/ps/83.4.544 15109052

[B30] Mariani P. Barrow P. A. Cheng H. H. Groenen M. M. Negrini R. Bumstead N. (2001). Localization to chicken chromosome 5 of a novel locus determining salmonellosis resistance. *Immunogenetics* 53 786–791. 10.1007/s00251-001-0387-7 11862411

[B31] Pal S. Dey S. Batabyal K. Banerjee A. Joardar S. N. Samanta I. (2017). Characterization of *Salmonella* Gallinarum isolates from backyard poultry by polymerase chain reaction detection of invasion (invA) and *Salmonella* plasmid virulence (spvC) genes. *Vet. World* 10 814–817. 10.14202/vetworld.2017.814-817 28831228 PMC5553153

[B32] Parmar D. Davies R. (2007). Fowl typhoid in a small backyard laying flock. *Vet. Rec.* 160:348. 10.1136/vr.160.10.348 17351184

[B33] Parsons B. N. Humphrey S. Salisbury A. M. Mikoleit J. Hinton J. C. Gordon M. A. (2013). Invasive non-typhoidal *Salmonella* typhimurium ST313 are not host-restricted and have an invasive phenotype in experimentally infected chickens. *PLoS Negl. Trop. Dis.* 7:e2487. 10.1371/journal.pntd.0002487 24130915 PMC3794976

[B34] Poultry Health Scheme Handbook (2013). *Poultry Health Scheme (PHS) Handbook*. Available at: www.defra.gov.uk

[B35] Prasad K. V. Ao Z. Yoon Y. Wu M. X. Rizk M. Jacquot S. (1997). CD27, a member of the tumor necrosis factor receptor family, induces apoptosis and binds to Siva, a proapoptotic protein. *Proc. Natl. Acad. Sci. U.S.A.* 94 6346–6351. 10.1073/pnas.94.12.6346 9177220 PMC21052

[B36] Purcell S. Neale B. Todd-Brown K. Thomas L. Ferreira M. A. Bender D. (2007). PLINK: a tool set for whole-genome association and population-based linkage analyses. *Am. J. Hum. Genet.* 81 559–575. 10.1086/519795 17701901 PMC1950838

[B37] Py B. Slomianny C. Auberger P. Petit P. X. Benichou S. (2004). Siva-1 and an alternative splice form lacking the death domain, Siva-2, similarly induce apoptosis in T lymphocytes via a caspase-dependent mitochondrial pathway. *J. Immunol.* 172 4008–4017. 10.4049/jimmunol.172.7.4008 15034012

[B38] Redmond S. B. Chuammitri P. Andreasen C. B. Palic D. Lamont S. J. (2011). Genetic control of chicken heterophil function in advanced intercross lines: associations with novel and with known *Salmonella* resistance loci and a likely mechanism for cell death in extracellular trap production. *Immunogenetics* 63 449–458. 10.1007/s00251-011-0523-y 21455609 PMC3111730

[B39] Revolledo L. (2018). Vaccines and vaccination against fowl typhoid and pullorum disease: an overview and approaches in developing countries. *J. Appl. Poult. Res.* 27 279–291 10.3382/japr/pfx066

[B40] Shivaprasad H. L. (2000). Fowl typhoid and pullorum disease. *Rev. Sci. Tech.* 19 405–424. 10.20506/rst.19.2.1222 10935271

[B41] Steele-Mortimer O. Knodler L. A. Marcus S. L. Scheid M. P. Goh B. Pfeifer C. G. (2000). Activation of Akt/protein kinase B in epithelial cells by the *Salmonella* typhimurium effector sigD. *J. Biol. Chem.* 275 37718–37724. 10.1074/jbc.M008187200 10978351

[B42] Taniguchi T. Harada H. Lamphier M. (1995). Regulation of the interferon system and cell growth by the IRF transcription factors. *J. Cancer Res. Clin. Oncol.* 121 516–520. 10.1007/BF01197763 7559730 PMC12201881

[B43] Thanh-Son T. Catherine B. Nigel S. Mark F. Pete K. Elisabeth Le B. D. (2012). A maximum likelihood QTL analysis reveals common genome regions controlling resistance to *Salmonella* colonization and carrier-state. *BMC Genomics* 13:198. 10.1186/1471-2164-13-198 22613937 PMC3428659

[B44] Tilquin P. Barrow P. A. Marly J. Pitel F. Plisson-Petit F. Velge P. (2005). A genome scan for quantitative trait loci affecting the *Salmonella* carrier-state in the chicken. *Genet. Sel. Evol.* 37 539–561. 10.1186/1297-9686-37-6-539 16093014 PMC2697224

[B45] Weerasooriya K. Fernando P. S. Liyanagunawardena N. Wijewardena G. Wijemuni M. I. Samarakoon S. (2017). Natural resistance of Sri Lankan village chicken to *Salmonella* gallinarum infection. *Br. Poult. Sci.* 58 644–648. 10.1080/00071668.2017.1376034 28868900

[B46] Wigley P. (2017). *Salmonella enterica* serovar Gallinarum: addressing fundamental questions in bacteriology sixty years on from the 9R vaccine. *Avian Pathol.* 46 119–124. 10.1080/03079457.2016.1240866 27791403

[B47] Wigley P. Hulme S. D. Bumstead N. Barrow P. A. (2002). In vivo and in vitro studies of genetic resistance to systemic salmonellosis in the chicken encoded by the SAL1 locus. *Microbes Infect.* 4 1111–1120. 10.1016/S1286-4579(02)01635-0 12361910

[B48] Yamaoka K. Saharinen P. Pesu M. Holt V. E. III Silvennoinen O. O’shea J. J. (2004). The Janus kinases (Jaks). *Genome Biol.* 5:253. 10.1186/gb-2004-5-12-253 15575979 PMC545791

[B49] Yoshida I. Azuma M. (1992). Function, molecular structure and gene expression of interferons. *Nihon Rinsho* 50 1845–1853. 1279237

[B50] Zhou X. Stephens M. (2014). Efficient multivariate linear mixed model algorithms for genome-wide association studies. *Nat. Methods* 11 407–409. 10.1038/nmeth.2848 24531419 PMC4211878

