# Supplementary material for: The Genomic Architecture of Fowl Typhoid Resistance in Commercial Layers
Source: Front Genet. 2018 Nov 19;9:519. doi: 10.3389/fgene.2018.00519 (PMC6252313; doi:10.3389/fgene.2018.00519)

**Supplemental Figure 1.** Quantitative bacterial counts on selective Brilliant Green Agar from the spleen (A) and liver (B) of *Salmonella enterica* serovar Gallinarum isolates from experimentally infected layer chickens. Data are shown as counts from individual animals with the median. These data show the outbreak (NI) isolate causes systemic infection to similar levels of the well characterised isolates 287/91 and 9.


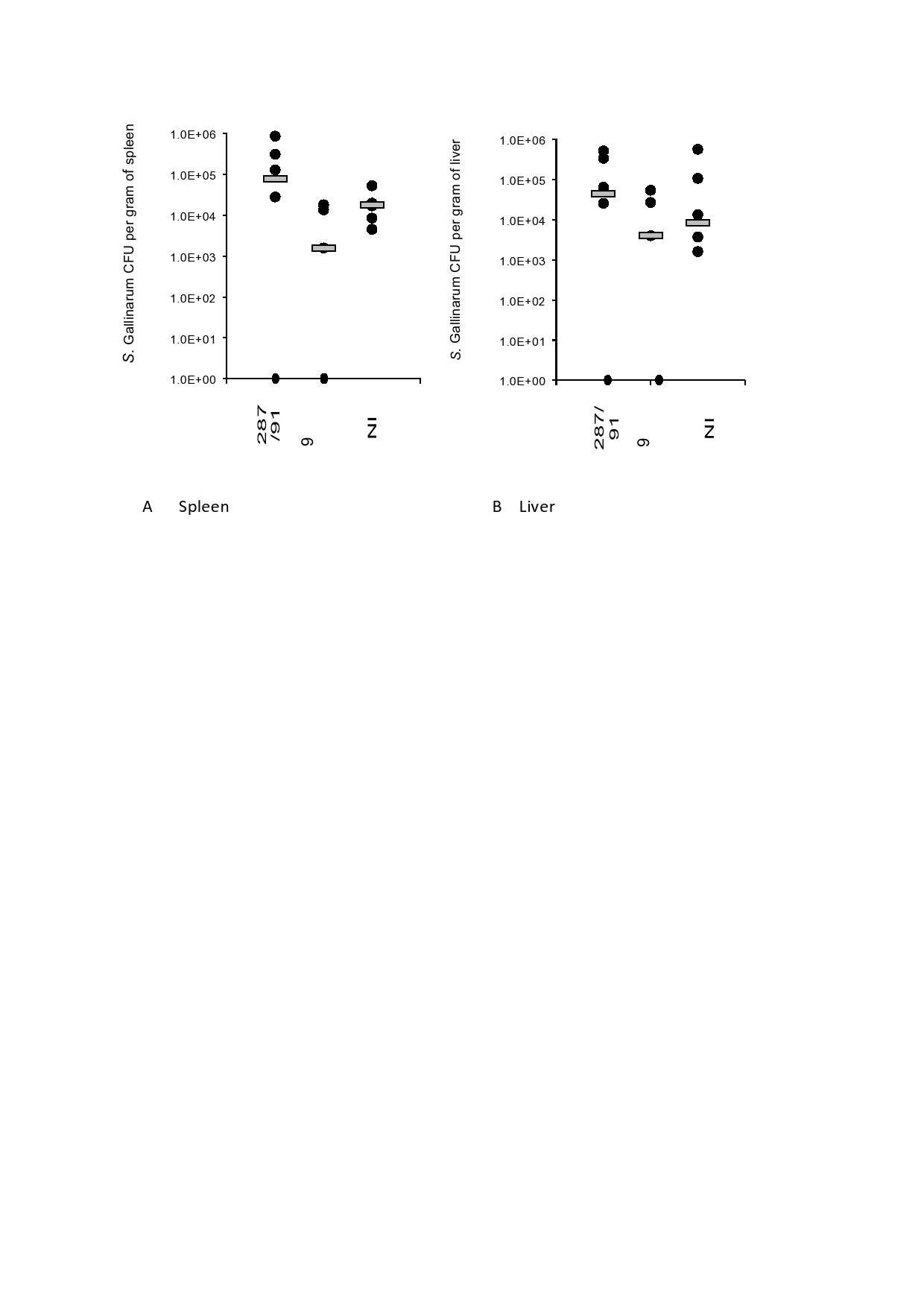

Supplement: Supplementary file 4 [file Data_Sheet_1.DOCX]
